# A Novel Feature Extraction Approach Using Window Function Capturing and QPSO-SVM for Enhancing Electronic Nose Performance

**DOI:** 10.3390/s150715198

**Published:** 2015-06-29

**Authors:** Xiuzhen Guo, Chao Peng, Songlin Zhang, Jia Yan, Shukai Duan, Lidan Wang, Pengfei Jia, Fengchun Tian

**Affiliations:** 1 College of Electronics and Information Engineering, Southwest University, Chongqing 400715, China; E-Mails: swugxz@163.com (X.G); pengchaocg@163.com (C.P.); z574066616@163.com (S.Z.); duansk@swu.edu.cn (S.D.); ldwang@swu.edu.cn (L.W.); jiapengfei200609@126.com (P.J.); 2 College of Communication Engineering, Chongqing University, Chongqing 400044, China; E-Mail: FengchunTian@cqu.edu.cn

**Keywords:** feature extraction, electronic nose, MWFC, QPSO, SVM

## Abstract

In this paper, a novel feature extraction approach which can be referred to as moving window function capturing (MWFC) has been proposed to analyze signals of an electronic nose (E-nose) used for detecting types of infectious pathogens in rat wounds. Meanwhile, a quantum-behaved particle swarm optimization (QPSO) algorithm is implemented in conjunction with support vector machine (SVM) for realizing a synchronization optimization of the sensor array and SVM model parameters. The results prove the efficacy of the proposed method for E-nose feature extraction, which can lead to a higher classification accuracy rate compared to other established techniques. Meanwhile it is interesting to note that different classification results can be obtained by changing the types, widths or positions of windows. By selecting the optimum window function for the sensor response, the performance of an E-nose can be enhanced.

## 1. Introduction

An electronic nose (E-nose) is a device composed of an array of gas sensors combined with a corresponding artificial intelligence algorithm. It is able to imitate the olfactory system of humans and mammals and is used for the recognition of gases and odors. Nowadays it plays a more and more important role in many fields, including odor analysis [1,2], product quality testing (such as food [3,4], tobacco [5], fermentation products [6], flavorings [7], *etc.*), disease diagnosis [8,9,10], environmental control [11,12], explosives detection [13], *etc.*

Previous work has confirmed that it is feasible to use an E-nose to detect bacteria, including the investigation of volatile organic compounds (VOCs) from cultures and swabs taken from patients with infected wounds [14,15,16]. However, it is still a great challenge for us to extract features from the original signals of sensors to further improve the accuracy of the pattern recognition. Firstly, we can extract features from the original response curves of sensors, such as peak values, integrals, differences, primary derivatives, secondary derivatives, adsorption slopes, and maximum adsorption slope at a specific interval from the response curves [17]. Independent component analysis (ICA) [18,19,20] is a statistical method for transforming an observed multidimensional vector into components that are statistically as independent from each other as possible. In this way, it removes the redundancies of the original data. Orthogonal signal correction (OSC) [21,22,23] is a new and popular data processing technique, and its basic idea is to remove information in the input matrix which is orthogonal to the target matrix. Principal component analysis (PCA) [24,25] extracts the important information from the observations which are inter-correlated and expresses this information as a set of new orthogonal variables called principal components. Secondly, we can also extract features based on some transformations, such as Fourier transformation and wavelet transformation, and then the transformation coefficients are used as features. The fast Fourier transformation (FFT) [26] gives useful information for rotating components since well-defined frequency components are associated with them. Wavelet transformation [27] is an extension of FFT. It maps the signals into new space with basis functions quite localizable in time and frequency space. The wavelet transform decomposes the original response into the approximation (low frequencies) and details (high frequencies). It bears a good anti-interference ability for the following pattern recognition to use the wavelet coefficients of certain sub-bands as features. 

For E-nose pattern recognition, a number of classifier algorithms have been widely used such as back propagation neural network (BPNN) [28], radical basis function neural network (RBFNN) [29] and support vector machine (SVM) [30]-based methods. Heuristic and bio-inspired methods [31], in particular, such as genetic algorithms (GA) [32], simulated annealing algorithm (SAA) [33], particle swarm optimization (PSO) [34] and recently the quantum-behaved particle swarm algorithm (QPSO) [35] have been applied for feature selection, sensor array optimization, and classifier parameter selection. The QPSO algorithm has been investigated in detail and it has been proved that the QPSO algorithm is a form of contraction mapping that can converge to the global optimum [36,37]. Ordinary optimization methods, which we mention in this paper, can easily to fall into a local minimum point and the QPSO outperforms them in the rate of convergence and convergence ability for many applications. SVM is a new machine learning method introduced by Vapnik [38,39] based on the small sample statistical learning theory. It adopts the structural risk minimization (SRM) principle, and finds the best compromise between the learning ability and the complexity of the model to get the best generalization ability according to the limited sample information. Because of its excellent learning, classification ability, high generalization capability and good ability of dealing with high dimensionality space, SVM has already been widely used with excellent performance in pattern recognition, function regression and density estimation problems in recent years. Ordinary classifiers based on empirical risk minimization principle, such as artificial neural networks, usually have the problem of over-fitting and are liable to fall into local minima. SVM can solve small-sample, non-linear and high dimension problems which use the structural risk minimization principle instead of empirical risk minimization.

Previous methods for feature extraction do not include the steady-state and transient information of the entire response curve. Moreover, a features-based transform domain will miss the time domain information and cannot completely reflect the characteristics of the entire response process. Extraction of features only using the response signal itself of an electronic nose cannot reflect the interaction between the array signal and other specific functions, which can provide more interesting information. In this paper, a novel feature extraction approach which can be referred to as moving window function capturing (MWFC) is introduced to enhance the performance of E-noses. In the rest of this paper, we will firstly introduce the sampling experiments in Section 2; then the whole methodology of MWFC with the QPSO based synchronization optimization of sensor array and SVM model parameters will be described in Section 3; the results and discussion will be shown in Section 4; finally we will draw our conclusions in Section 5.

## 2. Sampling Experiments

### 2.1. Material and Gas Sensor Array

Twenty SD (Sprague-Dawley) male rats, 6–8 weeks old and 225–250 g weight, were provided by the Experimental Animal Center of Daping Hospital, Third Military Medical University. All rats were randomly divided into four groups (five animals in each), including one control group and three groups infected by *Pseudomonas aeruginosa*, *Escherichia coli*, and *Staphylococcus aureus*, respectively. After the rats were anaesthesized, a small incision (about 1 cm long) was made in the hind leg in each rat. Then 100 μL of bacterial solution (10^9^ CFU/mL, *Pseudomonas aeruginosa*, *Escherichia coli*, or *Staphylococcus aureus*) was added into the wound described above in the respective infection group. Meanwhile, the same volume of physiological saline (0.9% *NaCl* solution) was added in the control group. The rats were used for the further experiment after 72 h. All experiments were approved by the Animal Care and Ethics Committee of Third Military Medical University.

**Figure 1:**
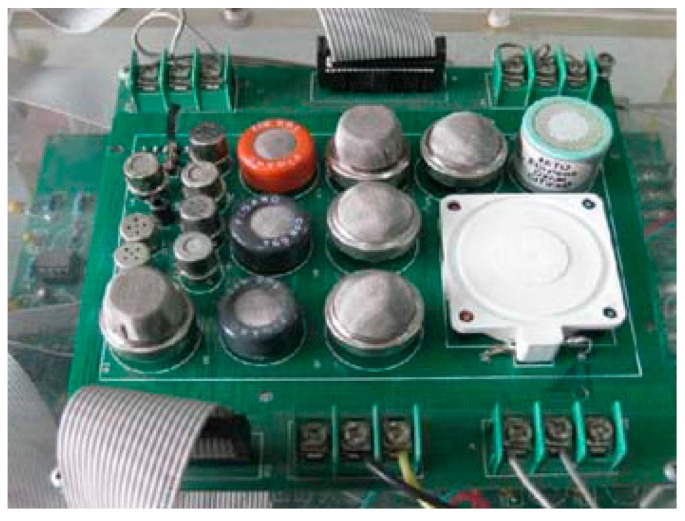
Sensor array.

The metabolites in the reproduction process of the three pathogens are shown in Table 1. According to the pathogen metabolites in Table 1 and the sensitive characteristics of gas sensors, fourteen metal oxide sensors and one electrochemical sensor are selected to construct the sensor array (shown in Figure 1). They are nine TGS sensors (TGS2600, TGS2602, TGS2620, TGS800, TGS822, TGS825, TGS826, TGS813, TGS816) from Figaro Engineering Inc. (Tianjin, China), one WSP-2111 XSC sensor from New Creators Electronic Technology Co. Ltd. (Shenzhen, China), two MQ sensors (MQ135, MQ138) from Winsen Electronics Technology Co. Ltd. (Zhengzhou, China), one QS-01 sensor from Bluemoon Technology Co. Ltd. (Shenzhen, China), one SP3S-AQ2 FIS sensor from FIS Inc. (Itami, Japan), and one AQ electrochemical sensor from Dart Sensors Ltd. (Exeter, UK). 

**Table 1:** Pathogens in wound infection and their metabolites.

Pathogens	Metabolites
*Pseudomonas aeruginosa*	Pyruvate, 2-nonanone, 2-undecanone, toluene, 1-undecene, 2-aminoacetophenone, esters, dimethyl disulfide, 2-heptanone, methyl ketones, dimethyl trisulfide, butanol, 2-butanone, sulphur compounds, isopentanol, isobutanol, isopentyl acetate
*Escherichia coli*	Ethanol, decanol, dodecanol, methanethiol 1-propanol,indole, methyl ketones, lactic acid, succinic acid, formic acid, butanediol, dimethyl disulfide, octanol, dimethyl trisulfide, acetaldehyde, hydrogen sulfide, formaldehyde, acetic acid, aminoacetophenone, pentanols
*Staphylococcus saureus*	Isobutanol, isopentyl acetate, ethanol, ammonia, 1-undecene, methyl ketones, 2-methylamine, 2,5-dimethylpyrazine, isoamylamine, trimethylamine, formaldehyde isopentanol, aminoacetophenone, acetic acid

**Figure 2:**
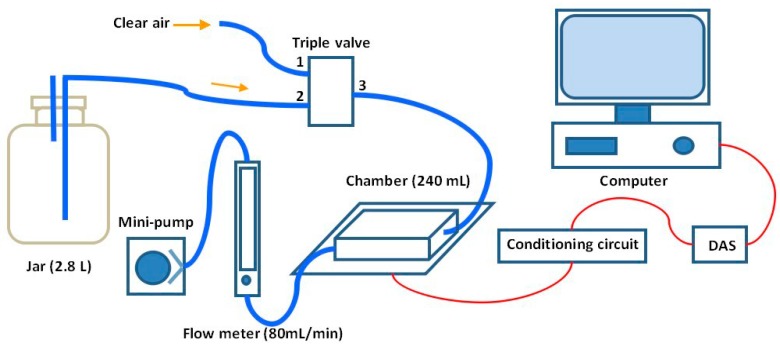
Schematic diagram of the experimental system.

The sensitive characteristics of the sensors used are listed in Table 2. All sensors are placed in a 240 mL stainless steel chamber which is coated with Teflon to avoid the attachment of VOCs. The schematic diagram of the experimental system is shown in Figure 2. A three-way valve is used to change the gas circuit to let the desired gas flow into the chamber. The flow velocity of gas is controlled by a flow meter and its value is set as 80 mL/min. A data acquisition system (DAS) is employed for the sensor signal sampling and its sample frequency is set as 10 Hz. The response of sensors is firstly processed by the conditioning circuit and then sampled and saved in a computer via the DAS.

**Table 2:** Response characteristics of gas sensors.

Sensors	Response Characteristics
TGS800	Methane, carbon monoxide, isobutane, hydrogen, ethanol
TGS813	Methane, propane, ethanol, isobutane, hydrogen, carbon monoxide
TGS816	Combustible gases, methane, propane, butane, carbon monoxide, hydrogen, ethanol, isobutane
TGS822	Organic solvent vapors, methane, carbon monoxide, isobutane, *n*-hexane, benzene, ethanol, acetone
TGS825	Hydrogen sulfide
TGS826	Ammonia, ethanol, isobutane, hydrogen
TGS2600	Gaseous air contaminants, methane, carbon monoxide, isobutane, ethanol, hydrogen
TGS2602	VOCs, odorous gases, ammonia, hydrogen sulfide, toluene, ethanol
TGS2620	Vapors of organic solvents, combustible gases, methane, carbon monoxide, isobutane, hydrogen, ethanol
WSP2111	Benzene, toluene, ethanol, hydrogen, formaldehyde, acetone
MQ135	Ammonia, benzene series material, acetone, carbon monoxide, ethanol, smoke
MQ138	Alcohols, aldehydes, ketones, aromatics
QS-01	VOCs, hydrogen, carbon monoxide, methane, isobutane, ethanol, ammonia
SP3S-AQ2	VOCs, methane, isobutane, carbon monoxide, hydrogen, ethanol
AQ	Carbon monoxide, methanol, ethanol, isopropanol, formaldehyde, acetaldehyde, sulfur dioxide, hydrogen, hydrogen sulfide, phenol, dimethyl ether, ethylene

### 2.2. Data Collection

**Figure 3:**
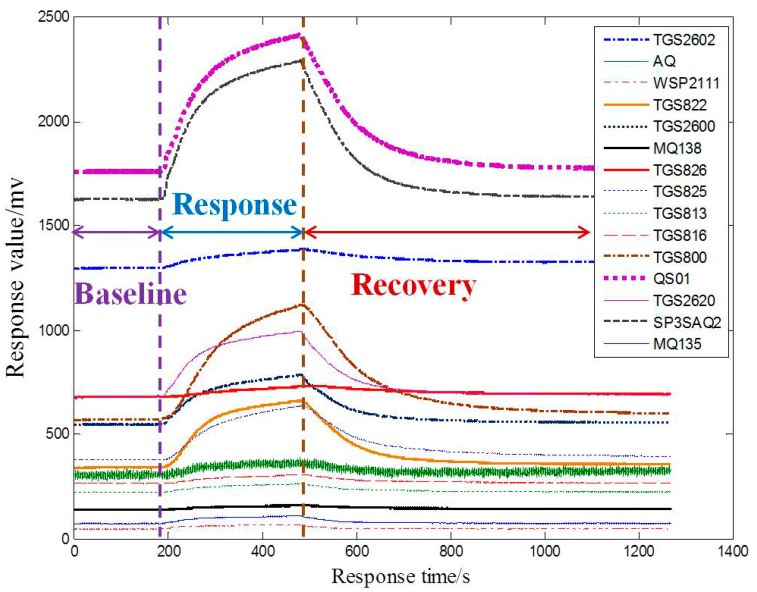
Response of E-nose to a wound infected with *P. aeruginosa.*

Each rat is placed in a jar with a volume of 2.8 L equipped with a rubber stopper. Two holes are made in the rubber stopper where two thin glass tubes were nserted, respectively. One glass tube is fixed above the wound as close as possible. The output gases of the tube which contains VOCs of the rat wound flow out of the bottle through the glass tube, and then flow into the test chamber through a Teflon tube. Clean air flows into the bottle through another glass tube. The dynamic headspace method is adopted during all the experiments, and the process is as follows: the first stage is the baseline stage, in which the sensors are exposed to clean air for three minutes. The second stage is the response stage, which the gas stream containing VOCs of the wound passes over the sensors for five minutes. The third stage is the recovery stage: the sensors are exposed to clean air again for fifteen minutes. At the end of each experiment, prior to the next experiment, a five minutes purging of the sensor chamber using clean air is performed. The gas flow is controlled by a gas flow rate control system, which contains a rotor flow meter, a pressure retaining valve, a steady flow valve and a needle valve. The flow rate is kept at 80 mL/min. Twenty experiments for each kind of rats in the same conditions are made, and so 80 samples are collected. The sensor response curves for one wound infected with *P. aeruginosa* are shown in Figure 3.

## 3. Methodology

### 3.1. Moving Window Function Capturing

In this work, a window is placed to different stage of the whole response and then the area values of two curves surrounded can be obtained by Newton-Cotes as follows:

(1) $$\int_{a}^{b}f(x)\mathrm{dx}\approx (b-a)\sum_{i=0}^{n}C_{i}^{\left(n\right)}f(x_{i})$$

where f(x) is integrand, [a,b] is integral interval and $C_{i}^{\left(n\right)}$ is Cotes coefficient. When n=4, $C_{0}^{4}=\frac{7}{90},C_{1}^{4}=\frac{16}{45},C_{2}^{4}=\frac{2}{15},C_{3}^{4}=\frac{16}{45},C_{4}^{4}=\frac{7}{90}$ and then Equation (1) is:

(2) $$\int_{a}^{b}f(x)\approx \frac{b-a}{90}[7f(x_{0})+32f(x_{1})+12f(x_{2})+32f(x_{3})+7f(x_{4})]$$

**Figure 4:**
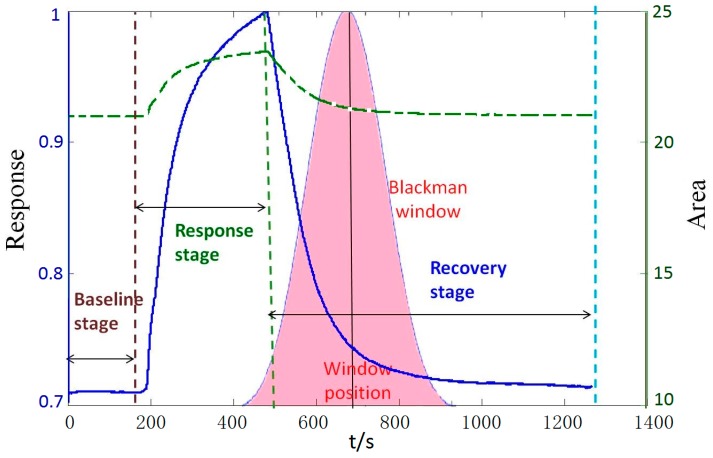
The schematic diagram of WFC technique.

**Figure 5:**
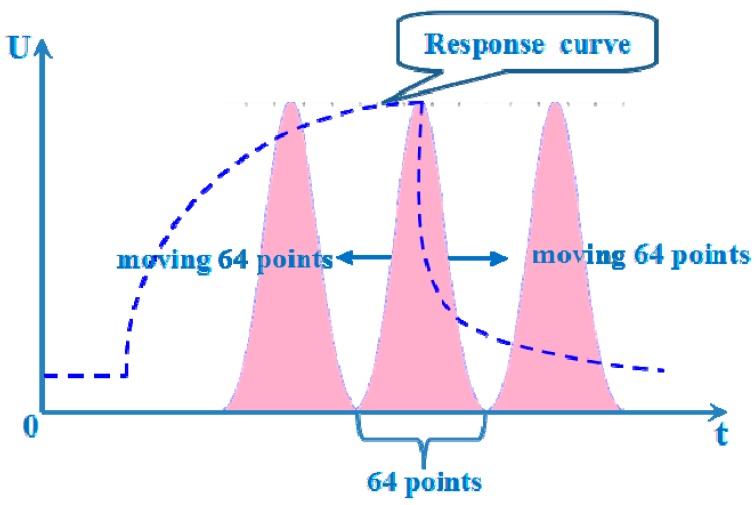
The schematic diagram of MWFC.

Then we can choose the value of the area surrounded by two curves as extracted features and refer to this method as window function capturing (WFC). The schematic diagram of the feature extraction approach using WFC is shown in Figure 4. The advantage of WFC is that it can be employed as a filter to capture information from the time domain rather than spectral representations. There are several kinds of common window functions, as shown in Table 3, and the performance of the E-nose will be changed by changing the width, position, shape of the window. In addition, we make the window move along with the time axis and simultaneously choose the area values of two curves during the moving process as features, which is referred to as moving window function capturing (MWFC). We place a 64 points window around the peak value and then make the window move 64 points to the left and right along with the time axis, respectively. Thus three area values surrounded by two curves can be obtained during the moving process and we can choose the three area values as features simultaneously. The schematic diagram of this method referred as MWFC is shown in Figure 5.

**Table 3:** Several kinds of common window functions.

Window	Equation (N is the Width of the Window)
Triang	$W(n)=\{\begin{array}{l} \;\frac{n}{\left(N/2\right)},n=0,1,2,...,N/2\\ W(N-n),n=N/2,...,N-1 \end{array}$
Blackman	$W(n)=0.42-0.5\cos(2\pi \frac{n-1}{N-1})+0.08\cos(4\pi \frac{n-1}{N-1}),n=0,1,2,...,\text{N}-1$
Hamming	$W(n)=0.54-0.46\cos(\frac{2\pi n}{N}),n=0,1,2,...,\text{N}-1$
Hanning	$W(n)=0.5-0.5\cos(\frac{2\pi n}{N}),n=0,1,2,...,\text{N}-1$
Boxcar	$W(n)=\{\begin{array}{c} 1,0\leq n\leq N-1\\ 0,else \end{array}$
Gaussian	$W(n)=e^{-\frac{1}{2}{\left[3(\frac{2n}{N-1})\right]}^{2}},n=0,1,2,...,\text{N}-1$

### 3.2. SVM

SVM is a new machine learning method introduced by Vapnik based on the small sample statistical learning theory [18,19]. Because of its high generalization capability and good ability to deal with high dimensionality space, SVM has already been widely used in pattern recognition, function regression and density estimation problems in recent years, with excellent performance.

The basic theory of SVM is to map the n-dimensional input vectors into K-dimensional feature space usually of K > n using a non-linear transformation φ(x) and then construct the optimal separating hyper-plane in the feature space:

(3) $$\begin{array}{ll} {\text{maxmise}}_{\text{α}} & \;W(\text{α})=\sum_{i=1}^{l}{\text{α}}_{i}-\frac{1}{2}\sum_{i,j=1}^{l}{\text{α}}_{i}{\text{α}}_{j}y_{i}y_{j}(x_{i}\cdot x_{j})\\ \text{subject to} & \;\sum_{i=1}^{l}{\text{α}}_{i}y_{i}=0,\;{\text{α}}_{i}\geq 0,\;i=1,...,l \end{array}$$

From Karush-Kuhn-Tucker complementarity condition, ${\text{α}}_{i}$ are not equal to zero only for the points nearest to the hyper-plane and ${\text{α}}_{i}$ corresponding to other points are zero. These points with non-zero ${\text{α}}_{i}$ are called support vectors because the hyper-plane is decided only by them, while the other points with ${\text{α}}_{i}=0$ are irrelevant. The discriminant function of classifying new points x is given by Equation (4):

(4) $$f\left(x\right)=w\cdot x+b=\sum_{i=1}^{l}y_{i}{\text{α}}_{i}\cdot (x_{i}\cdot x)+b$$

If the data vector x fulfils the condition f(x)>0, it will be classified into one class and when f(x)<0 it will be in the opposite class. If the original data are non-linearly separable and more complex separating surfaces are need, the non-linear SVM first maps the input data into a higher dimensional space called feature space by using a non-linear transformation φ, where the previous criterion can be implemented. Instead of calculating the inner products between the transformed data in the feature space, the inner products can still be measured in the original space with the introduction of the kernel function. Calculate the optimization problem in the feature space defined by kernel function implicitly, and Eqution (5) is transformed into:

(5) $$\begin{array}{ll} {\text{maxmize}}_{\text{α}} & \;W(\text{α})=\sum_{i=1}^{l}{\text{α}}_{i}-\frac{1}{2}\sum_{i,j=1}^{l}{\text{α}}_{i}{\text{α}}_{j}y_{i}y_{j}K(x_{i}\cdot x_{j})\\ \text{subject to} & \;\sum_{i=1}^{l}{\text{α}}_{i}y_{i}=0,\;0\leq {\text{α}}_{i}\leq C,\;i=1,...,l \end{array}$$

where $K(x_{i},x_{j})=\text{φ}(x_{i})\cdot \text{φ}(x_{j})$ is a kernel function which allows the inner products in feature space to be calculated directly in original space, without performing the mapping. The constant *C*, which can be regarded as regularization constant, is a positive number and determines the balance between accuracy on the training set and margin width. Increasing *C* leads to the more complex model structure and giving more importance to the errors on the training set in determining the optimal hyper-plane; decreasing *C* means smaller significance of the learning errors and simpler model structure with larger separation margin. Then we can construct optimal separating hyper-plane $y=\sum_{i=1}^{l}y_{i}{\text{α}}_{i}K(x_{i},x)+b$ in feature space. 

### 3.3. QPSO

Particle swam optimization (PSO) is a population-based swam intelligence algorithm that has attracted widespread interest from a large number of researchers. As a branch of PSO, quantum-behaved particle swarm optimization (QPSO), which was inspired by the thought of quantum mechanics and traditional PSO, shines for its simplicity, easy implementation, and fine search ability.

In the standard PSO model, with M particles in D-dimensional problem space, the position for particle *i* at iteration *t* can be represented as $X_{i}={\left(x_{i1}^{t},x_{i2}^{t},...,x_{iD}^{t}\right)}^{T},i=1,2,...,M$, the velocity for particle *i* at iteration *t* can be described as $V_{i}={\left(v_{i1}^{t},v_{i2}^{t},...,v_{iD}^{t}\right)}^{T},i=1,2,...,M$. By calculating the values of fitness function of M particles, the local optimal position (the position giving the best fitness value) of particle *i* at iteration *t* is recorded and represented as $pbest_{i}^{t}={\left(p_{i1}^{t},p_{i2}^{t},...,p_{iD}^{t}\right)}^{T}$. The global best position in the population at iteration *t* is represented as $gbest_{g}^{t}={\left(p_{g1}^{t},p_{g2}^{t},...p_{gD}^{t}\right)}^{T}$, where g is the index of the best particle among all the particles in the population. The velocity and position of particle *i* at iteration *t* + 1are update by the following equations:

(6) $$v_{id}^{t+1}=\text{ω}v_{id}^{t}+c_{1}r_{1}(pbest_{id}^{t}-x_{id}^{t})+c_{2}r_{2}(gbest_{id}^{t}-x_{id}^{t}),d=1,2,...,D$$

(7) $$x_{id}^{t+1}=x_{id}^{t}+v_{id}^{t+1},d=1,2,...,D$$

where *I* = 1, 2,…, *M*, *c*_1_ and *c*_2_ are learning factors, in general, *c*_1_ = *c*_2_ = 2 , *r*_1_ and *r*_2_ are random numbers uniformly distributed in [0,1], and ω is inertia weight which balances and reconciles the global and local searching capability.

QPSO was inspired by analysis of the convergence of the traditional PSO and quantum systems. In QPSO, we hypothesize that each particle is in a quantum state and is formulated by its wave function ψ(X,t) instead the position and velocity which are used in PSO. The probability density of a particle’s appearance in a certain position can be obtained from ${\left|\text{ψ}(X,t)\right|}^{2}$, and then the probability distribution function can be obtained. For the probability distribution function, through Monte Carlo stochastic simulation method, the particle’s position is updated according to the following equation:

(8) $$x_{id}^{t+1}=p_{id}^{t}\pm \text{α}\left|mbest_{id}^{t}-x_{id}^{t}\right|\times \ln(\frac{1}{u}),u=rand(0,1)$$

(9) $$p_{id}^{t}=\text{ϕ}\times pbest_{id}^{t}+(1-\text{ϕ})\times gbest_{id}^{t},\text{ϕ}=rand(0,1)$$

where α is the parameter of the QPSO algorithm, called contraction-expansion coefficient. We set the parameters as α=0.5+0.5×(loopcount−currentcount)/loopcount and $p_{id}^{t}$ is a local attractor, $mbest_{id}^{t}$ is the average optimal position of all the particles and defined as:

(10) $$mbest_{i}^{t}=\frac{1}{M}\sum_{i=1}^{M}P_{i}=(\frac{1}{M}\sum_{i=1}^{M}P_{i1},\frac{1}{M}\sum_{i=1}^{M}P_{i2},...,\frac{1}{M}\sum_{i=1}^{M}P_{id},)$$

**Figure 6:**
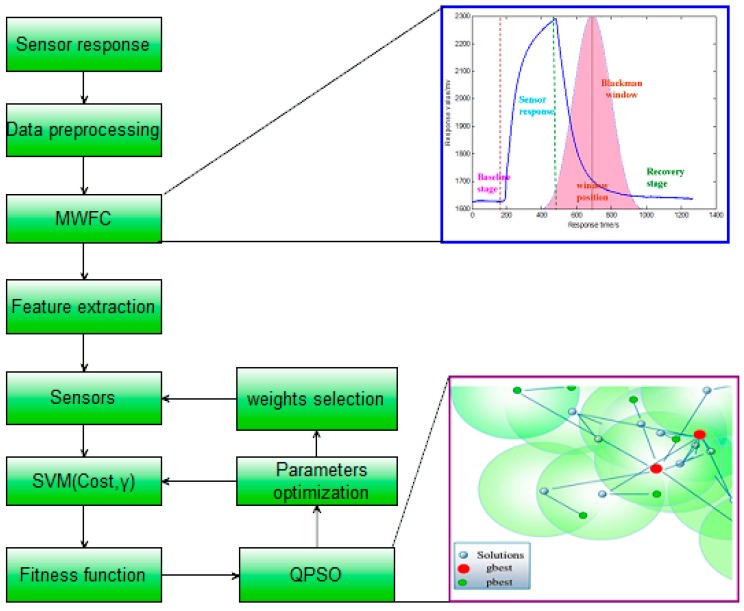
Flow chart of the optimization process.

Here, QPSO is implemented in conjunction with SVM for the classification of four different types of pathogens of rats wound infection, the flow chart of the optimization process is shown as Figure 6. 

### 3.4. Comparing Methods

To prove the efficiency of MWFC, we compare the accuracy rate between this method and some other feature extraction techniques combined with QPSO-SVM, such as peak value, rising slope, descending slope, FFT, DWT and WFC. Brief descriptions of these feature extraction methods are given in Table 4.

**Table 4:** Brief description of the parameters extracted from the sensor response.

Method	Description
Peak value	Max value of sensor response ${\text{ψ}}_{i}^{\max}$
Rising slope	$S_{R}=\frac{{\text{ψ}}_{i}^{\max}-Baseline}{T_{1}}$, *T_1_* is the time from the beginning of the adsorption stage to peak value.
Descending slope	$S_{F}=\frac{{\text{ψ}}_{i}^{\max}-Baseline}{T_{2}}$, *T_2_* is the time from peak value to the end of the desorption stage.
FFT	Coefficients of the DC component and first order harmonic component
DWT	Approximation coefficientsWavelet function is db5 wavelets and decomposition level 13.
WFC	The area value of sensor response curve and window curve surrounded
MWFC	The three area values of sensor response curve and window curve surrounded during the window moving process

## 4. Results

The window function of 64 time-points is placed at four different positions which response time are 180 s (the end of baseline stage), 330 s (the middle of response stage), 480 s (the end of response stage) and 930 s (the middle of the recovery stage), respectively, and four area values are extracted as different features. Table 5 shows the classification accuracy rate of six different windows placed at four different positions, respectively. It is observed that the type and position of the window function will both influence the classification. Compared with other positions, 480 s is a more suitable position relatively, where the Triang window, Blackman window, Hamming window, Hanning window, Boxcar window and Gaussian window can achieve classification accuracies of 95.0%, 92.5%, 92.5%, 92.5%, 90.0% and 95.0% , which are higher than the other positions. 

**Table 5:** Classification accuracy (%) of four positions based on different windows.

Windows	Positions			
	180 s	330 s	480 s	930 s
Triang	85.0	90.0	95.0	90.0
Blackman	82.5	90.0	92.5	87.5
Hamming	85.0	87.5	92.5	90.0
Hanning	85.0	90.0	92.5	90.0
Boxcar	80.0	90.0	90.0	87.5
Gaussian	85.0	92.5	95.0	87.5

Table 6 shows the classification accuracy of different windows placed at the 480 s position with different widths. It is interesting to note that the classification accuracy rate will be different as the width of the window is changing. It is observed that the width of 64-points is a relatively more suitable width compared to the other widths, whereby the Boxcar window obtains a classification of 90.0%, the Blackman window, Hamming window and Hanning window obtain classification rates of 92.5%, and the Triang window and Gaussian window can obtain a classification rate of 95%.

**Table 6:** Classification accuracy (%) of different windows shaped different widths.

Windows	Widths					
	32-points	64-points	128-points	256-points	512-points	1024-points
Triang	92.5	95.0	92.5	92.5	90.0	90.0
Blackman	87.5	92.5	92.5	90.0	90.0	87.5
Hamming	90.0	92.5	90.0	90.0	87.5	85.0
Hanning	90.0	92.5	92.5	90.0	90.0	87.5
Boxcar	87.5	90.0	90.0	87.5	87.5	85.0
Gaussian	90.0	95.0	92.5	92.5	92.5	90.0

**Figure 7:**
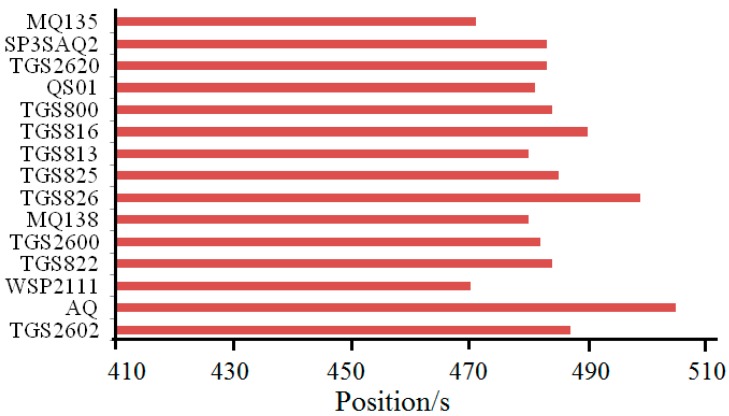
The positions where each sensor obtains the peak value.

From Table 5, it can be observed that 480 s is relatively a more suitable position compared with other positions. This means that the surrounding range of peak values contains much more key information to improve the classification accuracy. The positions where each sensor obtains its peak value are different, which is shown in Figure 7. Moreover, we take the width of window into consideration and find that the width of 64-points is relatively a more suitable width compared to the other widths shown in Table 6. 

**Figure 8:**
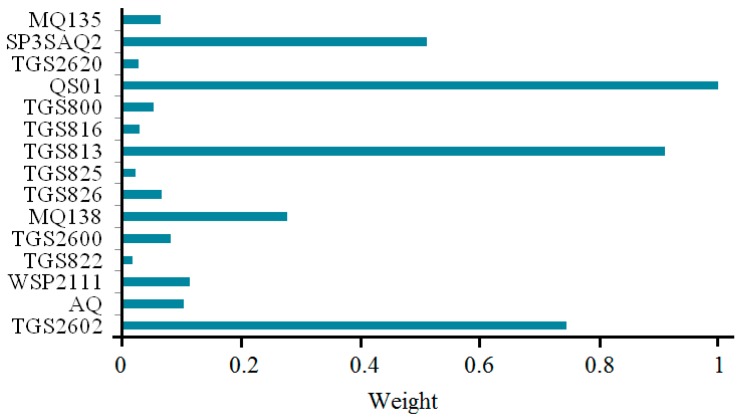
Optimal importance factors with QPSO for 15 sensors.

The importance of the 15 sensors is shown in Figure 8, where the corresponding optimal normalizing importance factors, that is the weighting coefficients of sensors, are [0.7445, 0.1032, 0.1144, 0.0180, 0.0816, 0.2771, 0.0668, 0.0224, 0.9095, 0.0299, 0.0539, 1.0000, 0.0279, 0.5109, 0.0646].

Table 7 shows the classification accuracy of MWFC with SVM and RBFNN. It is observed that the classification accuracy with SVM is higher than RBFNN and the classification accuracy with sensor optimization is higher than without sensor optimization. From Table 7 we can see that the QPSO-SVM method combined with weighting sensor array by importance factors obtains a 97.5% classification rate with the Triang window, 95.0% classification rate with the Blackman window, 95.0% classification rate with the Hamming window, 97.5% classification rate with the Hanning window, 95.0% classification rate with the Boxcar window, 97.5% classification rate with the Gaussian window. 

**Table 7:** Classification accuracy (%) of MWFC with SVM and RBF.

Methods	Types					
	Triang	Blackman	Hamming	Hanning	Boxcar	Gaussian
RBF-MWFC *^a^*	87.5	85.0	90.0	85.0	85.0	90.0
QPSO-RBF-MWFC *^b^*	92.5	87.5	92.5	90.0	87.5	90.0
SVM-MWFC *^a^*	92.5	90.0	92.5	92.5	92.5	92.5
QPSO-SVM-MWFC *^b^*	97.5	95.0	95.0	97.5	95.0	97.5

*^a^* means without sensor optimization and *^b^* with sensor optimization.

Table 8 lists the results of accuracy comparison of various feature extraction techniques. It is observed that the peak value method obtains an accuracy rate of 87.5%, the same as that of the rising slope, and is better than that of descending slope, which is only 85.0%. FFT and DWT achieve classification accuracies of 90.0% and 92.5%, and the SVM-WFC method which uses QPSO to optimize SVM parameters and the weights of each gas sensor can achieve an accuracy rate of 95.0%. It is interesting to note that the performance of the E-nose can be improved further when choosing the method of SVM-MWFC, which can achieve an accuracy rate of 97.5%.

**Table 8:** Accuracy comparison of various feature extraction techniques (%).

Feature Extraction	Accuracy Rate
Peak value	87.5
Rising slope	87.5
Descending slope	85
FFT	90.0
DWT	92.5
WFC	95.0
MWFC	97.5

To demonstrate the generalization to other datasets of the proposed approach, we use the feature extraction method of MWFC to deal with another two experimental E-nose datasets: (1) MWFC has been applied to deal with the data of an E-nose which detects five odors: nonane, 2-propyl alcohol, heptanal, 1-phenylethanone, and isopropyl myristate, and the classification results are shown in Table 9. More details about the sample preparation experiments can be found in [40]; (2) MWFC has also been applied to deal with the data of an E-nose which detects six indoor air contaminants including formaldehyde (HCHO), benzene (C_6_H_6_), toluene (C_7_H_8_), carbon monoxide (CO), ammonia (NH_3_) and nitrogen dioxide (NO_2_) and classification results are also shown in Table 9. More details about the sample preparation experiments can be found in [41]. 

**Table 9:** Accuracy of various feature extraction techniques for other datasets (%).

Feature Extraction	Accuracy Rate	
	Dataset in [40]	Dataset in [41]
Peak value	85.33	82.11
Rising slope	88.00	80.49
Descending slope	82.67	81.30
FFT	89.33	83.74
DWT	90.67	86.17
WFC	92.00	89.43
MWFC	93.33	91.06

From Table 9, MWFC also achieves better classification results of than the compared feature extraction methods. This shows the generalized performance of the feature extraction method of MWFC with other datasets. The efficacy of this approach does not depend on a particular dataset. 

## 5. Discussion

We use one-way analysis of variance (ANOVA) to test whether the feature extraction methods have a significant influence on the classification accuracy rate and then the test results can be obtained by SPSS as shown as Table 10. A one-sample Kolmogorov-Smirnov test confirms that the distributions of each feature extracted follow normal (or Gaussian) distributions. It can be found that the value of the F statistic is 553.976, which is significantly greater than 1 and the significance value is 0. Given the level of significance α = 0.05, we can reject the null hypothesis and conclude that there is a significant difference of accuracy rates under different feature extraction methods.

**Table 10:** ANOVA Results.

	Sum of Squares	df	Mean Square	F	Significant
Between Groups	4.1817	6	0.6969	553.976	0
Within Groups	0.3434	273	0.0013		
Total	4.5251	279			

**Figure 9:**
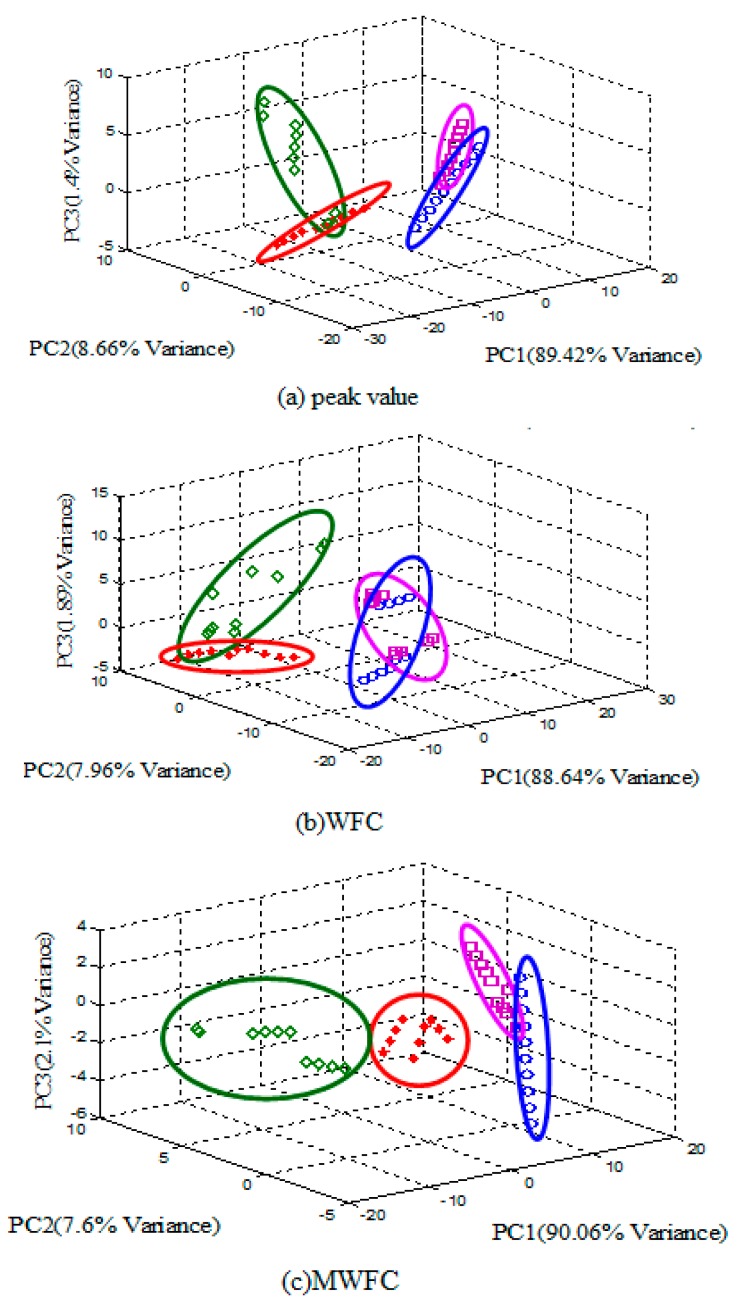
PCA score plot of different features.

To visualize the efficacy of the proposed method, PCA is applied for the peak value, WFC and MWFC features and the PCA score plots are shown from Figure 9, respectively. The higher degree of overlaps of four kinds of samples can be observed in Figure 9a and the distribution of four kinds of samples is relatively dispersive in Figure 9b, whereas, in Figure 9c, the cluster of four kind of samples are overlapping little and can be more easily distinguished. In a word, the performance of classification with the MWFC method is better than the others.

From the results shown above, the MWFC method can obtain a better accuracy rate for classification of different E-nose data than the compared methods. Peak value, which only represents the final steady-state feature of the entire dynamic response process in its final balance, reflects the maximum reaction degree change of sensors responding to odors. However, it misses all the transient response information of the reaction kinetics process and cannot describe the process well. Rising slope and descending slope also have specific physical meanings and represent the rate of the reaction of sensors responding to odors in the response and recovery stages, respectively. Although the rate of reaction of the sensors reflects the transient information in different stages, it only describes the reaction kinetics at one aspect. For the above features, it is difficult to distinguish tiny differences between response curves of different odors. They are not like the MWFC method which represents the cumulative total of the reaction degree change, accumulates these tiny differences in a specific way and makes these differences more significant. Moreover, these features only use the response signal itself and cannot reflect the interaction between the array signals to other specific functions, which can provide more interesting information. The widely used FFT, for which the basis functions are sine and cosine, maps the original data into a new space. It decomposes the original response into the superposition of the dc component and different harmonic components, and the feature characterized by amplitude of each component can be used for qualitative and quantitative analysis. However, FFT transforms the original signals from the time domain to the frequency domain and extracts features in the frequency domain. It misses the information in the time domain and cannot completely reflect the characteristics of the entire response process. Moreover, although extracting the coefficients of the dc component and first order harmonic component as features contains a large proportion of information of the original response curve, it misses the information in the higher harmonic components. Wavelet transform is an extension of the Fourier transform. It maps the signals into a new space with basis functions quite localizable in time and frequency space. DWT decomposes the original response into the approximation (low frequencies) and details (high frequencies). It bears good anti-interference ability for the followed pattern recognition to use the wavelet coefficients of certain sub-bands as features, so it obtains better result than the former features. 

However, extracting the approximation coefficients as features, which reflects the low frequencies information, misses the details, which reflect the high frequencies information, though the low frequencies signal contain much more information. What is more, there are many parameters of DWT to set, which have an effect on the decomposition results, and it is difficult to determine an optimal parameters set. WFC chooses the area surrounded by a window function curve and the original response curve as an extracted feature. Because the WFC method only places the window at the position of the peak value and extracts one area value as feature, it only reflects the information around the steady-state response of the entire dynamic response process in its final balance, which is the most important information to distinguish different types and concentrations of gases. It does not take a great deal of transient information in the whole response and recover stages into consideration and obtains worse results as compared to MWFC. 

MWFC is the extension of the WFC method, which can be employed as a filter to capture information from the time domain. It reflects the interaction between the response curve and different windows. If there are tiny differences between the response curves of different odors, the areas which are obtained by MWFC can accumulate these differences in a specific way, which is determined by different windows, and make these differences more significant. In this way, it can achieve a higher accuracy rate after selection of the proper window parameters.

## 6. Conclusions

In this paper, a novel feature extraction approach which can be referred to as moving window function capturing (MWFC) has been introduced and QPSO is implemented in conjunction with SVM for the classification of four different types of pathogens based on E-nose signals. The proposed approach has been compared with other established techniques for E-nose feature extraction, such as peak value, rising slope, descending slope, FFT, DWT and WFC. The results prove the efficacy of proposed method which can lead to an ideal accuracy rate for classification. It has also been shown that the performance of an E-nose will be enhanced by optimizing the SVM parameters and the gas sensor array. In addition, the types, widths or positions of windows will influence the classification result and better classification results can be obtained by choosing the appropriate type, width or position of windows.
